# A neural extracellular matrix-based method for *in vitro* hippocampal neuron culture and dopaminergic differentiation of neural stem cells

**DOI:** 10.1186/1471-2202-14-48

**Published:** 2013-04-18

**Authors:** Patricia García-Parra, Marcos Maroto, Fabio Cavaliere, Neia Naldaiz-Gastesi, José Iñaki Álava, Antonio G García, Adolfo López de Munain, Ander Izeta

**Affiliations:** 1 Tissue Engineering Laboratory, Department of Bioengineering, Instituto Biodonostia, Hospital Universitario Donostia, San Sebastian, 20014, Spain; 2 Neuroscience Area and CIBERNED, Instituto Biodonostia, Hospital Universitario Donostia, San Sebastian, 20014, Spain; 3 Instituto Teófilo Hernando de I + D del Medicamento. Departamento de Farmacología y Terapeútica and Servicio de Farmacología Clínica del IIS Hospital Universitario de La Princesa, Facultad de Medicina, Universidad Autónoma de Madrid (UAM), Madrid, 28029, Spain; 4 Department of Neuroscience and CIBERNED, University of Basque Country (UPV/EHU), Zamudio, 48170, Spain; 5 Basque Culinary Center R&D, San Sebastian, 20009, Spain

**Keywords:** Neural extracellular matrix, Subventricular zone, Neuronal culture, Neural progenitor cells, Dopaminergic differentiation

## Abstract

**Background:**

The ability to recreate an optimal cellular microenvironment is critical to understand neuronal behavior and functionality *in vitro*. An organized neural extracellular matrix (nECM) promotes neural cell adhesion, proliferation and differentiation. Here, we expanded previous observations on the ability of nECM to support *in vitro* neuronal differentiation, with the following goals: (i) to recreate complex neuronal networks of embryonic rat hippocampal cells, and (ii) to achieve improved levels of dopaminergic differentiation of subventricular zone (SVZ) neural progenitor cells.

**Methods:**

Hippocampal cells from E18 rat embryos were seeded on PLL- and nECM-coated substrates. Neurosphere cultures were prepared from the SVZ of P4-P7 rat pups, and differentiation of neurospheres assayed on PLL- and nECM-coated substrates.

**Results:**

When seeded on nECM-coated substrates, both hippocampal cells and SVZ progenitor cells showed neural expression patterns that were similar to their poly-L-lysine-seeded counterparts. However, nECM-based cultures of both hippocampal neurons and SVZ progenitor cells could be maintained for longer times as compared to poly-L-lysine-based cultures. As a result, nECM-based cultures gave rise to a more branched neurite arborization of hippocampal neurons. Interestingly, the prolonged differentiation time of SVZ progenitor cells in nECM allowed us to obtain a purer population of dopaminergic neurons.

**Conclusions:**

We conclude that nECM-based coating is an efficient substrate to culture neural cells at different stages of differentiation. In addition, neural ECM-coated substrates increased neuronal survival and neuronal differentiation efficiency as compared to cationic polymers such as poly-L-lysine.

## Background

The extracellular matrix (ECM) is responsible for the promotion of cell adhesion, proliferation and differentiation, and hence maintenance of tissue homeostasis throughout the whole organism. In the nervous system, neural ECM (nECM) components provide the necessary cues for the growth of neurons and neurites both *in vitro* and *in vivo*[1]. Even during embryonic development, nECM plays a major role in the formation and expansion of the neural crest [2,3]. Although mature nECM is amorphous, it also contains dense areas around neurons known as perineural networks (PNNs), which in turn leave spaces at the sites where synaptic contacts are established [4,5]. PNNs are mainly composed of proteoglycans, collagen, laminin, fibronectin and neuropeptides. The latter seem to be involved in the regulation of neuronal plasticity [6] as well as in neuroprotection [7].

Topographical and biochemical characteristics of the substrate might thus be critical for axonal outgrowth and regeneration of neural circuits *in vivo.* For instance, cell therapy trials of Parkinson’s disease indicate that the mere existence of a damaged microenvironment promotes the generation of new dopaminergic neurons, although perhaps in numbers insufficient for clinical improvement [8].

In the adult central nervous system (CNS), neurogenesis seems to be restricted to two specific areas: (i) the subventricular zone (SVZ) [9,10] and (ii) the subgranular layer of the dentate gyrus of the hippocampus [11,12]. Neural stem cells (NSCs) with multipotent potential can be derived from rodent SVZ [10,11,13]. *In vitro*, the differentiation of NSCs into neuronal or glial cells is induced in adherent culture conditions (with serum) after EGF and FGF2 withdrawal and by specific neuronal- and/or glial-inducing factors. As the differentiation process progresses, NSCs restrict their ability to self-renew and differentiate to other lineages.

In a previous article we described the development, characterization and functional validation of a nECM-based polymeric support used as a biocompatible and efficient *in vitro* microenvironment for neuronal differentiation of both PC12 cells and adult skin-derived precursor cells [14]. Since nECM proteoglycans are involved in axonal regeneration and in post-traumatic neuronal plasticity in the CNS [15,16], the rationale for the present methodological article was to use the nECM component of this polymeric support to study neuronal development and, in particular, dopaminergic differentiation. The presence of collagen, laminin, entactin and certain proteoglycans in the basal composition of nECM provides the signals that cells need to be anchored to the substrate and start differentiation. Moreover, hyaluronic acid enhances the hydration of the matrix, which in turn will facilitate cell movement and neurite extension along the neuronal differentiation progress as well as provide fixation sites for growth factors [17] and other glycosaminoglycan-binding molecules [18]. Finally, netrins support neuronal differentiation acting as cellular and neurite-chemoattractant factors, as it occurs in the naturally developing neural tissue [19].

As of today, several types of polymeric supports have been described to sustain neural cell adhesion and migration [20,21]. Recent works demonstrated that polymeric substrates sustain differentiation of stem/progenitor cells and help generate functional synapses because the spatial, mechanical and biochemical cues that neurons receive from the environment strongly determine their *in vitro* behaviour [22-25]. Nevertheless, these studies also suggest the importance of soluble factors released from the ECM. Therefore, culturing stem/progenitor cells on three-dimensional ECMs could be a successful approach to maintain multipotent progenitor cell cultures for longer *in vitro*, as well as to achieve higher percentages of neuronal differentiation. In the present work, we studied biocompatibility and neuronal differentiation of primary neural cells at different stages of development on nECM-coated substrates. We were particularly interested in improving primary neuronal cultures of embryonic rat hippocampal cells and to obtain higher levels of dopaminergic neurons from neonatal rat SVZ multipotent progenitor cells, as compared to more “classical” cell culture conditions such as poly-L-lysine (PLL) coating.

## Results and discussion

### Culture of rat hippocampal cells on neural extracellular matrix (nECM)-coated surfaces

**Figure 1:**
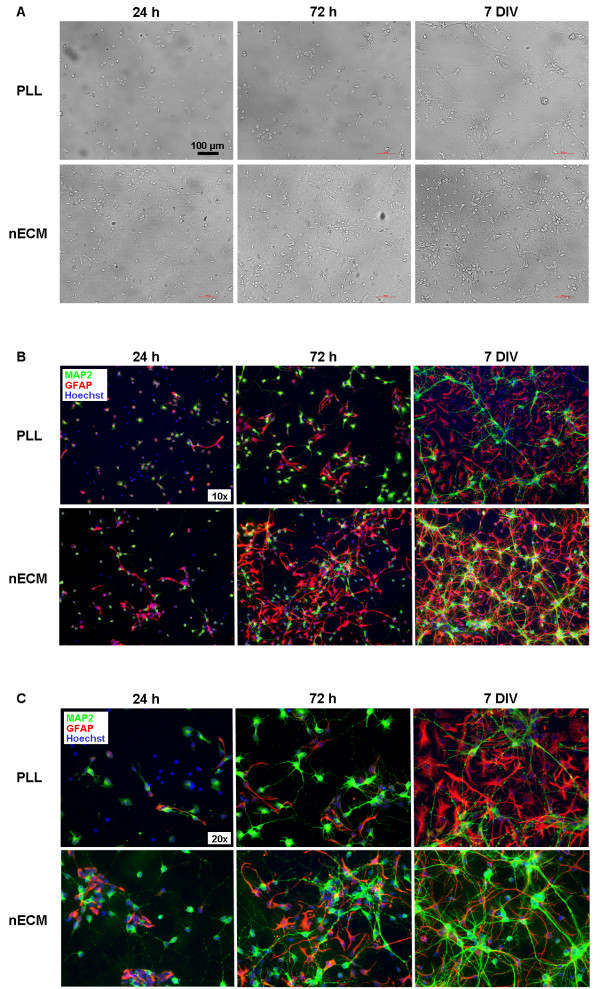
**Establishment of primary hippocampal cultures on PLL- and nECM-coated coverslips.** Hippocampal cells were seeded either on PLL- or nECM-coated coverslips and culture progression was observed on both substrates for 7 days. (**A**) Bright field microscopic images taken at 24 h, 72 h and 7 days of culture, respectively. Scale bars, 100 μm. (**B**) Immunofluorescence analyses (×10 objective). Neuronal and glial cells grown for 24 h, 72 h and 7 days on both substrates were fixed and immunolabelled with anti-MAP2 (green) and anti-GFAP (red) antibodies, respectively. Cell nuclei were counterstained with Hoechst (blue). (**C**) Magnification (×20 objective) of immunofluorescence images shown in panel B. All images are representative of three independent experiments.

**Figure 2:**
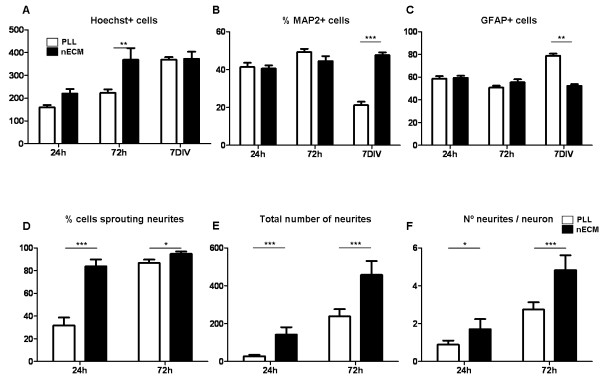
**Quantitative assessment of hippocampal neuronal and glial differentiation on PLL- and nECM-coated coverslips.** (**A**-**C**) To estimate the total cell number in each photograph, the number of nuclei stained by Hoechst was counted (**A**) and the percent of cells positive for MAP2 (**B**) and GFAP (**C**) immunostains were determined. (**D**-**F**) Focusing on MAP2 positive cells, the number of cells sprouting neurites (**D**) as well as the total number of neurites (**E**) was determined at 24 and 72 h, respectively. Next, the number of neurites per neuron was calculated (**F**). At least six different fields out of three independent experiments were quantified at each substrate and time point. Statistical analyses were done by using two-way ANOVA followed by Bonferroni’s posttests (*P < 0.05; ** P < 0.01; *** P < 0.001).

To evaluate the effect of nECM on primary hippocampal cultures as compared to more traditional cell substrates as PLL, E18 rat embryo hippocampi were extracted, dissected and cells seeded either on nECM- or PLL-coated coverslips. As expected, a neural network (neuronal plus glial) was progressively established during the first 7 days of culture on both PLL- and nECM-based substrates (Figure 1). Culture progression (in terms of cell fate determination and neuronal sprouting) was apparently faster on nECM than in PLL, as visualized by immunofluorescent detection of the mature neuronal marker MAP2 and the glial marker GFAP (Figure 1B-C). A proliferative advantage (as seen by Hoechst positive cell counts) was detected on nECM at early time points (p < 0.01; Figure 2A), which was apparently lost at day 7 of culture, presumably due to cells reaching confluence. Interestingly, the number of cells committed to the neuronal lineage (MAP2+ cells, Figure 2B) was similar on both substrates at day 1–3, while cells were actively growing. However the number of neurons was significantly higher (47.6 ± 3.5% vs 21.2 ± 4.6%, respectively; p < 0.001) on nECM than in PLL at day 7, when cultures were fully committed to differentiation. Furthermore, we observed the reverse effect (p < 0.01) on glial lineage-committed cells (GFAP+, Figure 2C), so it can be said that nECM promoted neuronal differentiation while PLL favoured glial commitment.

With regard to neuronal sprouting, after 24 and 72 h of culture on nECM, significant increases in the percentage of neurons sprouting neurites compared with their PLL counterparts were detected (Figure 2D). Moreover, these neurons extended a significantly higher total number of neurites (p < 0.001; Figure 2E) and a significantly higher (p < 0.001) number of neurites per neuron (4.8 ± 0.8 vs 2.8 ± 0.4 neurites per neuron at day 3, respectively; Figure 2F) than those on PLL-coated coverslips. Finally, nECM-based cultures were maintained for up to 30 days with cell media changed every 7 days (as compared to 2-day media changes in the case of PLL; data not shown). These data might support a role of netrins in cell survival as previously suggested by Tang and colleagues [26]. Moreover, since netrins are known to induce neuronal sprouting during embryonic development, this effect might also be observed in the adult and partially help to explain the differences in neurite number in the presence (nECM) or absence (PLL) of added netrins. Additionally, cationic polymers such as PLL may be easily digested by cells, whereas a natural and more complex polymeric support such as nECM should be more efficient in sustaining cell adhesion and, consequently, might improve cell survival overall.

**Figure 3:**
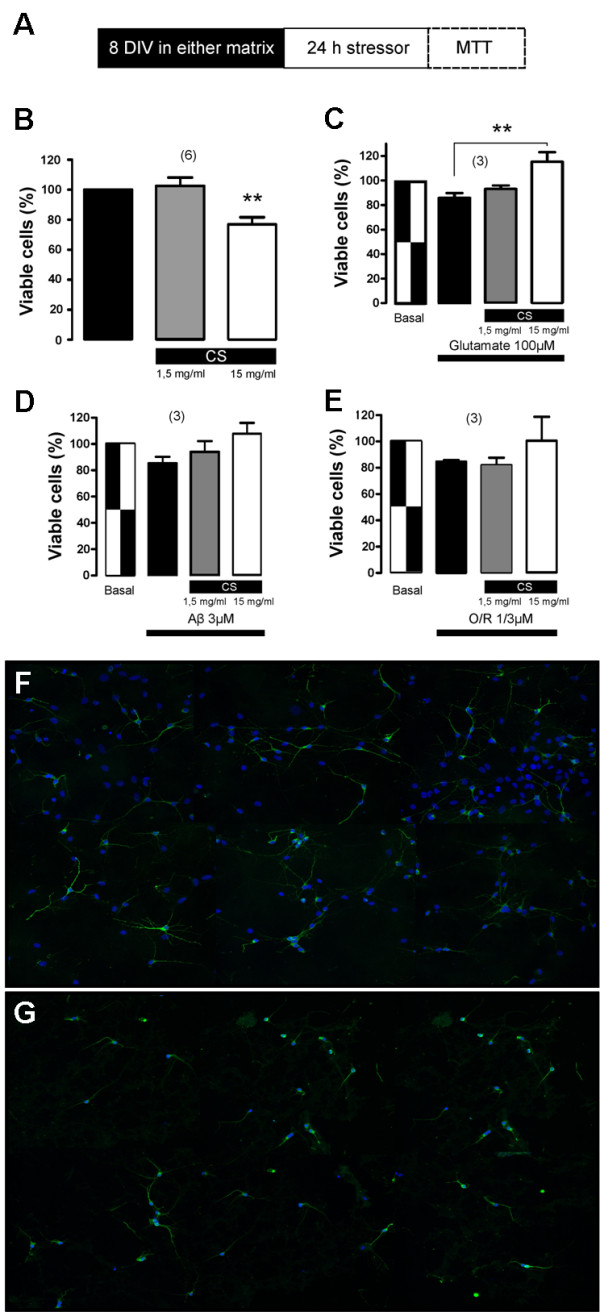
**Effect of chondroitin 4-sulfate (CS) incorporated to nECM on hippocampal cell viability.** (**A**) Experimental set up. After one week on CS-containing nECM and 24 hours of stressor treatment, hippocampus cell viability was measured by MTT assay. (**B**) Percentage of viability of hippocampal cells cultured on nECM in the absence (black bar) or presence of CS (1.5 mg/ml, grey bar; 15 mg/ml, white bar). (**C**) Percentage of viability of hippocampal cells cultured on nECM in the absence/presence of CS and in the absence/presence of 100 μM glutamate treatment. In each case, the treatments applied are described below bars. (**D**-**E**) Same as previous but this time in the presence of 3 μM amyloid beta 23:35 peptide (Aβ) (**D**) and 1 μM oligomycin plus 3 μM rotenone (O/R) (**E**) for 24 h. ** 0.001 < P < 0.01. The numbers between brackets on top of panels B-D show the number of independent experiments performed in each case. (**F**-**G**) Composite image containing immunofluorescence images of cells cultured on control nECM (**F**) and on 15 mg/ml CS-containing nECM (**G**). Cells were immunolabelled with anti-MAP2 antibody (green) and nuclei counterstained with Hoechst (blue).

As a proof-of-concept study to test usefulness of the hippocampal neuron culture system in drug testing, we set to examine the effect on neuronal viability of different molecules incorporated onto nECM. Chondroitin sulfate proteoglycans (CSPGs) are extracellular matrix molecules present in both developing and adult CNS, with a conflicting role in either promoting neural growth and plasticity or restricting them [15,27]. CSPGs are significantly upregulated in glial scar and are believed to be the main cause of regeneration failure after nervous system injury, through inhibition of neuronal growth cone formation [28-30]. However, other works describe a neuroprotecting role of chondroitin 4-sulfate (CS) as a PNN component [7,31]. To illuminate this controversy, hippocampal cells were seeded on nECM in the absence or presence of two different CS concentrations incorporated as a matrix component (1.5 and 15 mg/ml), and neuronal viability was measured by MTT assay at day 8 (Figure 3A). Somewhat unexpectedly, a significant (p < 0.01) decrease in cell viability was appreciated when higher CS concentration was used (Figure 3B). This reduction in cell viability was confirmed by anti-MAP2 immunofluorescence (Figure 3F-G). In addition, and as previously reported, CS concomitantly inhibited axonal sprouting and reduced axon growth. These data would be consistent with an anti-regenerative role of chondroitin 4-sulfate, as shown specifically for chondroitin 4-sulfate but not for chondroitin 6-sulfate [32].

Once tested in isolation, the ability of CS-incorporated nECM to protect neuronal culture from cytotoxic damage (or otherwise) was analyzed. To this end, known cytotoxic compounds such as glutamate, beta-amyloid 25:35 peptide (Aβ) and oligomycin-rotenone (O/R) were added to cultures grown on nECM in the absence or presence of previously tested CS concentrations (Figure 3C-E). Higher concentration of CS significantly (P < 0.01) increased viability of neurons that were treated with 100 μM glutamate for 24 h (Figure 3C). The same neuroprotective tendency of CS was observed for Aβ and O/R treatments, but no statistical significance was achieved even at higher CS dosing (Figure 3D-E). Thus, in the presence of a cytotoxic agent as glutamate, a molecule liberated by injured cells in the central nervous system, CS (and probably other proteoglycans present in nECM) seemed to play a neuroprotective role, reversing the reduction of cellular viability seen when cells were seeded onto control nECM. Its apparent neuroprotective effect may be explained due to the known capacity of chondroitin 4-sulfate to sequester toxic compounds added to the medium. Alternatively, CS might increase the expression of antioxidant enzymes [33] or modulate the immuno-inflammatory response [31]. These observations should be studied in more detail because they could have effects on promoting repair following spinal cord injury when the ECM changes its composition and expression of CS proteoglycans is augmented [34]. Following chondroitinase ABC treatment, many groups have reported some improvement in spinal cord injury models and *in vitro* neuronal cultures recreating glial scar ECM [35-37].

### Dopaminergic differentiation of subventricular zone (SVZ)-derived multipotent progenitor cells on nECM-coated surfaces

**Figure 4:**
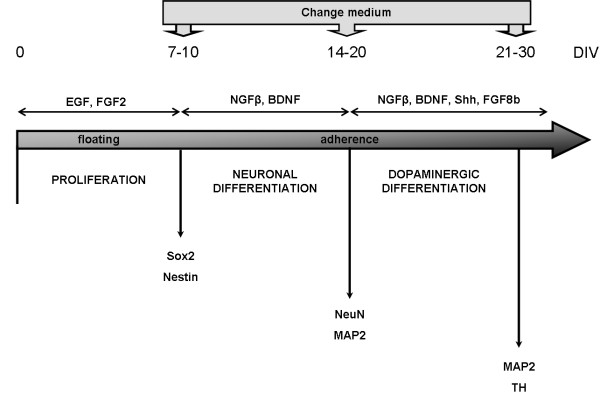
**Strategy for neuronal dopaminergic differentiation of SVZ-derived progenitor cells.** The scheme depicts the experimental approach to attain dopaminergic neurons from SVZ progenitors. Proliferation was conducted for 7–10 days in free-floating neurospheres, in the presence of EGF and FGF2. Neuronal differentiation was induced after adhesion to PLL- or nECM-coated coverslips in the presence of NGFβ and BDNF. Dopaminergic differentiation was observed after 35 days in neuronal differentiation conditions, in the presence of Shh and FGF8b. Immunofluorescence screening for different markers of neuronal differentiation (Sox2: 24 h; Nestin: 24 h and 7 DIV; NeuN: 14 DIV; TH: 21 DIV) was performed throughout the experiment with positivity arising at different days of differentiation as represented. DIV: days *in vitro* (in differentiation medium).

**Figure 5:**
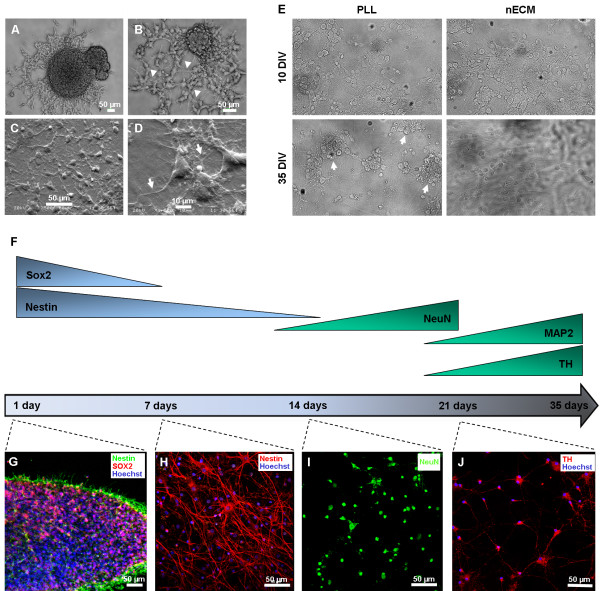
**Differentiation of SVZ-derived progenitors on PLL- and nECM-coated coverslips.** SVZ-derived neurospheres (at 7 days of proliferation) were seeded on either PLL- or nECM-coated coverslips. (**A-B**) Images taken by phase contrast microscopy at 24 (**A**) and 72 h (**B**) post-differentiation on nECM. Arrowheads in panel B point to the arising neuritic network. Scale bars, 50 μm. (**C**-**D**) Analysis by scanning electron microscopy (SEM) 20 days post-differentiation on nECM. Panel D arrows show matrix-embedded neurites. Scale bars, 50 and 10 μm, respectively. (**E**) Focusing on neurosphere differentiation after 10 or 35 days, cell degeneration (arrowheads) was observed only in the case of PLL substrates at 35 days in culture. (**F**) Representation of the appearance/disappearance of marker expression throughout differentiation culture. (**G**-**J**) Representative images of neurodevelopmental marker expression in SVZ-derived progenitors on nECM-coated coverslips. SVZ neurospheres differentiated on nECM in the presence of the factors shown in Figure 5 were analyzed by immunofluorescence with the following markers: (**G**) Sox2 (red), Nestin (green), Hoechst (blue); (**H**) Nestin (red), Hoechst (blue); (**I**) NeuN (green); (**J**) MAP2 (red), Hoechst (blue). All images are representative of three independent experiments.

To study a possible effect of nECM on progenitor cell differentiation, we selected a well-known neuronal differentiation protocol, starting up from multipotent progenitor cells of neonatal rat subventricular zone (SVZ). SVZ cells were isolated and expanded for 7–10 days as floating neurospheres by following previously described protocols [38], then adhered to PLL- or nECM-coated coverslips for an additional 7–10 days to yield differentiated but seemingly immature neurons (Figures 4 and 5). Figure 4 represents the differentiation procedure, which lasted a maximum of 30 days. First, immature neurons were derived for 7–10 days in the presence of neurotrophic factors NGFβ and BDNF (basic neuronal medium). Second, dopaminergic phenotype was induced by additional Shh and FGF8b administration (dopaminergic medium) for 7–10 days, as described [39-43].

The effect of nECM on neurosphere differentiation was evident as early as 24–72 h post-differentiation (Figure 5A-B), in the form of radial migration of cells arising from the centre of the neurospheres and subsequent formation of neurite networks (arrowheads in Figure 5B). Scanning electron microscopy analyses (Figure 5C-D) confirmed extended neuritogenesis at day 20 of differentiation, newly formed neurites being often embedded by non-degraded nECM (arrows in Figure 5D). This “embedding effect” was also appreciated in Z-stack confocal views of the samples (see below). These data suggested a mechanical and trophic effect of the nECM on neuronal differentiation. On the other hand, SVZ cells started to show picnotic nuclei characteristic of cells entering apoptosis at 35 DIV (arrows in Figure 5E), a phenomenon that was not observed in nECM-based cultures.

**Figure 6:**
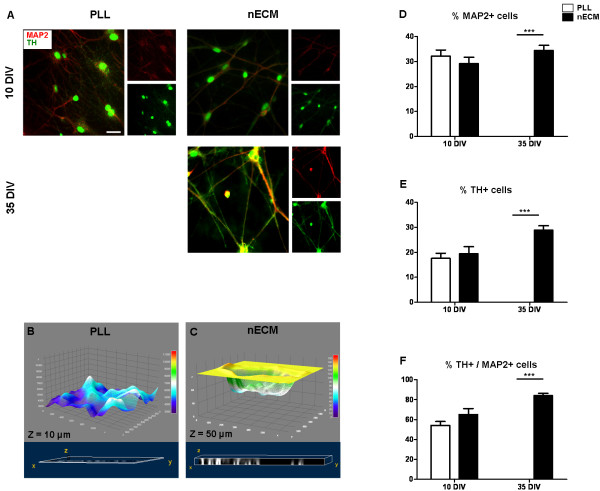
**Dopaminergic differentiation of SVZ-derived progenitors on PLL- and nECM-coated coverslips.** (**A**) SVZ-derived neurospheres were seeded both on PLL- and nECM-coated coverslips and differentiated in the presence of a dopaminergic medium (including NGFβ, BDNF, Shh and FGF8b) for 10 or 35 days, respectively. Double stainings were performed with anti-MAP2 (red) and anti-TH (green) antibodies and nuclei counterstained with Hoechst (not shown). Colocalization of both markers is shown in the Merged (large) panels. All images are representative of three independent experiments. Scale bar, 40 μm. (**B-C**) Z stack analysis of cells differentiated for 10 days in PLL (**B**) or nECM (**C**). Upper panels (grey) show cell profiles, demonstrating cell incorporation into the matrix; lower panels (blue) show stack as a volume with Z value as 10 μm in PLL or 50 μm in nECM (see also Additional file 1 Video S1 and Additional file 2 Video S2). (**D**-**F**) To estimate the percentage of MAP2 and TH positive cells in each substrate and time condition, MAP2 (**D**) and TH (**E**) positive cells were determined. To obtain the ratio of TH positive neurons (**F**), TH positive cell number was divided by the MAP2 positive cell number. At least six different fields out of three independent experiments were quantified in each medium, substrate and time point. Statistical analyses were done by using two-way ANOVA assay followed by Bonferroni’s posttests (*P < 0.05; ** P < 0.01; *** P < 0.001).

To further characterize progenitor cell differentiation, immunofluorescence analyses were performed for a number of neural markers at different times post-differentiation (Figure 5F). Immediately after the proliferation phase and prior to differentiation, cells expressed the multipotential progenitor markers Sox2 and Nestin, demonstrating lack of commitment to a more specified phenotype (Figure 5G). As expected, no expression of markers for mature neurons (NeuN, MAP2, TH) was detected at early time points. Nestin was highly expressed up to day 7 (Figure 5H)*,* decreased at day 14 and was undetectable at later time points (not shown). Starting from day 14, mature neuronal markers were detected. NeuN expression was observed from day 14 to 21 of differentiation (Figure 5I) and data not shown. Similarly, MAP2 was detected from day 21 to 35 of differentiation (not shown). Finally, dopaminergic neuronal differentiation, as assessed by the expression of tyrosine hydroxylase (TH) in MAP2+ cells, was detected in the same time window (Figure 5J and Figure 6A). Interestingly, confocal sectioning of PLL- and nECM-based cultures showed a marked difference in cell penetration of the substrate: while PLL-based cultures showed practically no depth in this analysis (Z = 10 μm), nECM-based cultures presented neuronal cells deeply embedded within the substrate, as shown by a five fold increase in the Z axis length (Z = 50 μm; Figure 6B-C and Additional file 1: Video S1 and Additional file 2: Video S2).

To quantify the differentiation of SVZ progenitors on both PLL- and nECM-based substrates, the numbers of cells positive for MAP2 and TH were counted. At early time points (day 10) the percentage of dopaminergic neurons was not significantly different (p > 0.05) on both substrates (54.2 ± 10.3 vs 65.1 ± 15.8% MAP2^+^TH^+^ cells on PLL and nECM, respectively; Figure 6D-F). In contrast, a significant increase of dopaminergic neurons (84.0 ± 6.3% MAP2^+^TH^+^ neurons on nECM as compared to none on PLL) was found after 1 month in culture (Figure 6D-F).

In summary, the fact that SVZ progenitor cells maintained their multipotentiality for a week and thereafter differentiated towards dopaminergic neuron lineage for up to 35 days allowed us to temporarily map the expression of several proteins related to neuronal development, in what might be viewed as a relevant *in vitro* system for the assessment of unanswered questions related to neural development.

## Conclusions

We have extended our previous observations on the ability of nECM to support *in vitro* neuronal differentiation, with the following achievements (i) we have recreated complex neuronal networks of embryonic rat hippocampal cells, and (ii) we achieved high levels of dopaminergic differentiation of SVZ progenitor cells. Furthermore, we showed proof-of-concept of the usefulness of this system to test the effect of added factors (as incorporated into the matrix) on neuronal behaviour. The biocompatible substrate enriched with nECM components represents a valid method to study neuronal development by using cells at different stages of commitment. Neural ECM-coated substrates increased culture maintenance window and neuronal differentiation efficiency as compared to cationic polymers such as PLL.

## Methods

### Animal care

All experiments were carried out in accordance with the guidelines established by the National Council on Animal Care and were approved by the local Animal Care Committee of the Universidad Autónoma of Madrid (UAM, Madrid, Spain) or the University of Basque Country (Spain) Animal Ethics committee, as relevant, following European Communities Council Directive of 22 September 2010 (2010/63/EU). Every possible effort was made to minimize animal suffering and the number of animals used.

### Isolation and culture of rat hippocampal cells

Pregnant Sprague–Dawley rats (Charles River Laboratories International, Inc., Wilmington, USA) were sacrificed by CO_2_ inhalation and 18-day embryos (E18) were immediately removed by caesarean section. Hippocampi were dissected rapidly by using a Leica CLX 150× stereomicroscope (Leica Microsystems, Barcelona, Spain) under sterile conditions in cold (4°C) PBS. The tissue was digested with 0.5 mg/ml papain (Sigma-Aldrich, Madrid, Spain) and 0.25 mg/ml DNAase (Sigma-Aldrich, Madrid, Spain). The enzymes were dissolved in a Ca^2+^- and Mg^2+^-free PBS solution containing 1 mg/ml BSA (Sigma-Aldrich, Madrid, Spain) and 10 mM glucose (Sigma-Aldrich, Madrid, Spain) at 37°C for 20 min. The papain solution was replaced with 5 ml Neurobasal™ A Medium (Life Technologies Corporation, Paisley, UK) supplemented with 2% B27 Supplement (Life Technologies Corporation, Paisley, UK), 1% L-glutamine solution 200 mM (Sigma-Aldrich, Madrid, Spain) and 1% penicillin/streptomycin solution (Sigma-Aldrich, Madrid, Spain). The digested tissue was gently triturated by suction using a glass pipette flamed on the tip to avoid cellular damage. The cell suspension was centrifuged for 4 min at 1,200 rpm. The supernatant was removed and the cells resuspended in 5 ml of medium and seeded on both PLL- and nECM-coated multiwell plates in the same maintenance medium at a density of 120,000 cells/ml. Cells were maintained in these conditions in a 5% CO2 incubator at 37°C.

### Isolation and differentiation of rat SVZ progenitor cells

Neurosphere cultures were prepared from 4 to 7-day-old Sprague–Dawley rat pups (Charles River Laboratories International, Inc., Wilmington, USA). Subventricular zone (SVZ) tissue from 2–3 rat brains were isolated and minced with a McIllwain tissue chopper (H. Saur Laborbedarf, Reutingen, Germany) and digested for 10 min at 37°C in 5 ml of 0.25% trypsin/EDTA solution (Sigma-Aldrich, Madrid, Spain). Digestion was stopped by adding an equal volume of trypsin inhibitor (Sigma-Aldrich, Madrid, Spain) and 0.01% DNAse I for 5 min at room temperature. Cell suspension was centrifuged for 10 min at 600 g and the pellet was mechanically dissociated 25 times in NeuroCult medium (Stem Cell Inc., Grenoble, France) using a glass Pasteur pipette and 20 times using 1 ml pipette tips. Non-dissociated cells were decanted and the single cell suspension was counted using the Neubauer method. Cells were seeded in proliferation medium: NeuroCult with 10% neural stem cell factors (both from Stem Cell Inc., Grenoble, France), 1% L-glutamine solution 200 mM (Sigma-Aldrich, Madrid, Spain), 1% penicillin-streptomycin solution (Sigma-Aldrich, Madrid, Spain), 20 ng/ml EGF and 10 ng/ml FGF2 (both from Promega, Madrid, Spain), at a density of 10,000 cells/cm^2^ and cultivated in suspension for 7 days at 37°C with 5% CO_2_. EGF and FGF2 were added fresh every 2–3 days.

The differentiation of neurospheres on 12 mm-diameter PLL- and nECM-coated coverslips (10 neurospheres/coverslip) was carried out in Neurobasal™ A Medium supplemented with 2% B27 Supplement and 1% N2 Supplement (all from Life Technologies Corporation, Paisley, UK), 10% FBS (LGC Standards S.L.U., Barcelona, Spain), 1% L-glutamine solution 200 mM and 1% penicillin-streptomycin solution (both from Sigma-Aldrich, Madrid, Spain). Every two days, half of the medium was replaced and supplemented gradually and at different time periods up to 35 days with 50 ng/ml rat recombinant nerve growth factor β (NGFβ) (Sigma-Aldrich, Madrid, Spain), 50 ng/ml human recombinant brain derived neurotrophic factor (BDNF) (Sigma-Aldrich, Madrid, Spain), 200 ng/ml human recombinant Sonic hedgehog (Shh) (R&D, Minneapolis, USA) and 200 ng/ml mouse recombinant fibroblastic growth factor 8b (FGF8b) (R&D, Minneapolis, USA).

### Development of neural extracellular matrix (nECM)-coated subtrates

Neural extracellular matrix (nECM)-coated substrates were developed as described [14]. Briefly, 12 mm-diameter coverslips were incubated with a solution of Cultrex® basement membrane extract (BME) without phenol red (8.32 mg/ml; Trevigen, Gaithersburg, USA; it is a soluble form of basement membrane purified from Engelbreth-Holm-Swarm tumor; the major components of this BME include laminin, collagen IV, entactin, and heparin sulfate proteoglycan), hyaluronan of low molecular weight (31 kDa, HLMW) (7.5 mg/ml; R&D, Minneapolis, USA), Netrin-G1a and Netrin-4 (25 μg/ml; R&D, Minneapolis, USA) in phosphate-buffered saline (PBS) (Life Technologies Corporation, Paisley, UK). For some experiments, chondroitin 4-sulfate glycosaminoglycan (Sigma-Aldrich, Madrid, Spain) was added to the mix together with hyaluronic acid. In order to polymerize and adsorb the matrix on the exposed glass surface they were kept for 24 h at 37°C in sterile conditions. Finally, excess PBS was eliminated and coverslips left to dry up for 1 h under laminar flow and UV light. As a control, PLL-coated coverslips were prepared by incubation of 12 mm-diameter coverslips with a 0.01% solution of poly-L-lysine (Sigma-Aldrich, Madrid, Spain) in distilled water, followed by PBS washing and air drying under laminar flow for 1 h.

### Immunofluorescence and confocal microscopy analyses

Differentiating cells were washed with PBS and fixed in 4% paraformaldehyde solution (Electron Microscopy Sciences, Hatfield, UK) for 20 min at room temperature. Cells were further washed with PBS and permeabilized for 1 hour in 0.3% Triton® X-100 (Sigma-Aldrich, Madrid, Spain) in PBS (PBS-T) and 5% normal donkey serum (Sigma-Aldrich, Madrid, Spain). After that, cells were incubated with the appropriate primary antibody diluted in PBS-T for 2 h at room temperature. Primary antibodies used were anti-Sox2 rabbit polyclonal (used at 1:1,000 dilution, Millipore #AB5603, Billerica, USA), anti-nestin mouse monoclonal IgG1 (1:200, Millipore #MAB353, Billerica, USA), anti-NeuN mouse monoclonal IgG1 (1:200, Millipore #MAB377, Billerica, USA), anti-GFAP mouse monoclonal IgG1 (1: 200; Dako, #M0761, Denmark), anti-MAP2 mouse monoclonal IgG1 (1:200; Millipore #MAB3418, Billerica, USA) and anti-tyrosine hydroxilase rabbit polyclonal (TH; 1:1,000; Millipore #AB152, Billerica, USA) antibodies. After 3 washes (5 min each) fixed cells were incubated with the appropriate secondary antibody diluted in PBS-T for 1 h at room temperature. Secondary antibodies used were donkey anti-mouse Alexa Fluor® 488 (1:1,000) and donkey anti-rabbit Alexa Fluor® 546 (1:1,000) (both from Life Technologies Corporation, Paisley, UK). Prior to embedding in Mowiol® mounting medium (Sigma-Aldrich, Madrid, Spain), cells were counterstained with 10 μg/ml Hoechst 33258 (Sigma-Aldrich, Madrid, Spain) for 5 min and washed with distilled water. Fluorescence images were obtained by using a Nikon Eclipse E600 FN microscope (objectives 10× and 20×) coupled to Nikon Digital Sight and analysed with Nikon NIS-Elements. Confocal images were generated with a Zeiss LSM 510 microscope coupled to Zeiss Axion camera and analyzed with Zeiss ZEN image analysis software (2008; SP1.1; Carl Zeiss MicroImaging, S.L., Barcelona, Spain). Z stack was analyzed with the plug in 3D viewer of Image J software. In both cases, at least ten different fields out of three independent experiments (n = 3) were quantified.

### Quantification of cell viability with the MTT assay

Cell viability was assessed by quantitative colorimetric assay with (3-(4,5-Dimethylthiazol-2-yl)-2,5-diphenyltetrazolium bromide (MTT), as previously described [44]. Briefly, a final concentration of 0.5 mg/ml MTT labelling reagent (Sigma-Aldrich, Madrid, Spain) in Neurobasal™ A Medium was added to each well containing embryonic hippocampal cells cultured for 8 days on control nECM- and chondroitin 4-sulfate containing nECM-coated coverslips, and incubated for 2 h. After that, colorimetric determination of MTT reduction was measured at 540 nm with a Kontron Instruments UVIKON 922 spectrophotometer. These experiments were repeated after treatment of embryonic hippocampal cells with several cytotoxic agents such as glutamate, oligomycin-rotenone (O/R) and amyloid beta 25:35 (Aβ) (all from Sigma-Aldrich, Madrid, Spain) for 24 h. These experiments were performed with cells from day 7 to day 8 in culture. For statistical analyses, cells in each nECM-coated well in absence of toxic were taken as 100% of viability.

### Scanning electron microscopy analyses

For Scanning Electron Microscopy (SEM) analyses, substrates with cells were fixed with Vitrosec® 70 (Panreac Química S.A.U., Barcelona, Spain) for 20 min and stained with May-Grünwald’s eosin methylene blue solution (Merck, Madrid, Spain). Samples were dried and mounted onto aluminium disks and coated with gold-argon. Imaging was performed on a JEOL JSM-5910-LV scanning electron microscope using a 20 kV acceleration voltage. Samples were analyzed by an INCA-300 energy dispersed system (EDS) coupled to the microscope.

### Statistical analyses

Data are expressed as the mean ± SD of the number of independent experiments (n). Statistical significance of the results was assessed by using GraphPad Prism software (version 5.01 for Windows). Two-way analysis of variance with subsequent pairwise multiple comparison procedures (Bonferroni’s posttests) was used to assess statistical significance between means. The statistical significance (*) was established at P-values <0.05 (** P < 0.01; *** P < 0.001).

## Abbreviations

nECM: Neural-like extracellular matrix; PLL: Poly-L-lysine; SVZ: Subventricular zone; Shh: Sonic hedgehog; PNNs: Perineural networks; NSCs: Neural stem cells; HLMW: Hyaluronan of low molecular weight; TH: Tyrosine hydroxilase.

## Competing interests

The authors declare that they have no competing interests.

## Authors’ contributions

PGP and FC conceived the project, carried out the cellular studies, and analyzed all the experiments with contribution of its design from JIA and AGG. MM and NNG performed the pharmacological and immuno-morphological studies under the supervision of AGG and PGP, respectively. PGP, MM, AGG and FC drafted the manuscript while ALM and AI revised it critically for important intellectual content. FC, JIA, AGG, ALM and AI acquired funding necessary for the completion of the study. All authors read and approved the final manuscript.

## Supplementary Material

Additional file 1: Video S1 Three-dimensional analysis of SVZ-derived progenitors differentiated on PLL-coated substrates for 10 days. A 3D projection of 40 consecutive confocal sections obtained from PLL-coated coverslips is shown. The depth of imaging was 14.6 microns with a thickness (interval) of 0.374 microns between sections. Video edition was done with ZEN 2008 and ImageJ software. Magnification: 480X. Click here for file

Additional file 2: Video S2 Three-dimensional analysis of SVZ-derived progenitors differentiated on nECM-coated substrates for 10 days. A 3D projection of 40 consecutive confocal sections obtained from nECM-coated coverslips is shown. The depth of imaging was 14.98 microns with a thickness (interval) of 0.384 microns between sections. Video edition was done with ZEN 2008 and ImageJ software. Magnification: 480X. Click here for file
